# Comparative genomic analyses reveal key traits for biocontrol and the promotion of plant growth in *Paenibacillus* strains

**DOI:** 10.1007/s11274-026-04811-6

**Published:** 2026-02-23

**Authors:** Luciano Nascimento de Almeida, Mirelly Jady Fernandes e Silva, Blenda de Freitas Rodrigues Jesuino, Sumaya Martins Tupy, Jorge Henrique Resende Vieira, Gabriela Amaral Xavier, João Paulo Lopes da Rocha, Osiel Silva Gonçalves, Mateus Ferreira Santana

**Affiliations:** 1 Grupo de Genômica Eco-Evolutiva Microbiana, Laboratório de Genética Molecular de Microrganismos, Departamento de Microbiologia, Instituto de Biotecnologia Aplicada À Agropecuária, Universidade Federal de Viçosa, Minas Gerais, Brazil; 2 Department of Biological Science, Universidade Estadual Do Centro-Oeste (Unicentro), Paraná, Brazil

**Keywords:** Biological control, Secondary metabolites, Phytohormones, Sustainable agriculture, Bioinformatics

## Abstract

*Paenibacillus* species have emerged as promising candidates for sustainable agriculture due to their functional versatility in plant growth promotion and biocontrol. We performed a comparative genomic analysis of 428 high-quality *Paenibacillus* genomes to assess their ecological adaptability and biotechnological potential. The analyzed strains originated from diverse environments, reflecting broad ecological distribution. Functional annotation revealed a widespread occurrence of plant growth-promoting traits (PGPTs), including phosphate and potassium solubilization, siderophore biosynthesis, nitrogen fixation, and phytohormone-related compounds. On average, the genomes contained 249 genes associated with biofertilization, 190 with phytohormone production, 97 linked to bioremediation, and around 322 involved in competitive exclusion. The open pan-genome configuration (*b* = 0.503) highlights notable genetic plasticity and ongoing gene acquisition. While the core genome was enriched in essential functions, accessory and unique fractions carried genes associated with environmental adaptation and niche specialization. Analysis of mobile genetic elements (MGEs) showed that some PGPT-related genes occur in mobile regions, suggesting horizontal gene transfer contributes to the dissemination of beneficial traits. Diverse BGCs, including those encoding Bacillopaline, Tridecaptin, Fusaricidin B, Paeninodin, and Polymyxin, were identified, many with antimicrobial potential. CAZyme profiling revealed abundant chitinases, supporting pathogen suppression capacity. No virulence factors were detected, and antibiotic resistance genes were rare, underscoring the genus’ low pathogenicity. Altogether, these findings position *Paenibacillus* as a genetically and functionally diverse group with strong potential as a safe, sustainable resource for developing biofertilizers and biopesticides.

**Supplementary Information:**

The online version contains supplementary material available at 10.1007/s11274-026-04811-6.

## Introduction

*Paenibacillus* is a genus of Gram-positive, facultatively anaerobic, endospore-forming bacteria comprising at least 240 species (Dai et al. 2019; Xie et al. 2016). Initially classified under the genus *Bacillus*, these bacteria were reclassified as a distinct genus in 1993 following a comprehensive taxonomic reassessment (Ash et al. 1993). Members of *Paenibacillus* exhibit remarkable biochemical and morphological diversity and inhabit a wide array of environments, including soil (McSpadden Gardener 2004), rhizospheres (Xie et al. 2012), marine sediments (Ravi et al. 2007), clinical specimens (Ouyang et al. 2008), and fermented foods (He et al. 2007), among others. Frequent isolation of *Paenibacillus* from the rhizosphere, particularly from agriculturally important crops (Raza et al. 2008), underscores the ecological versatility of the genus, reflecting its broad adaptation to diverse environmental niches (Huang et al. 2020).

Over the past two decades, growing attention has been directed towards the ecological significance and biotechnological potential of *Paenibacillus* species (Lal and Tabacchioni 2009). Numerous species have been recognized as plant growth-promoting rhizobacteria (PGPR), exerting both direct and indirect effects on plant development. Direct mechanisms include the biosynthesis of phytohormones, phosphate solubilization, and biological nitrogen fixation. Indirectly, plant growth is stimulated through the suppression of phytopathogens via the secretion of antimicrobial metabolites and hydrolytic enzymes (Soni et al. 2021). Several studies have demonstrated the efficacy of *Paenibacillus* species in promoting growth across various crops. For instance, the *P. polymyxa* CR1 strain has been shown in greenhouse trials to enhance the growth of maize, potato, and cucumber. This strain also exhibited in vitro nitrogen fixation, phosphate solubilization, and production of indole-3-acetic acid (IAA) (Weselowski et al. 2016). Similarly, *Paenibacillus* sp. YSY-4.3 was found to synthesize phytohormones (IAA, gibberellic acid, and zeatin), form biofilms and siderophores, and exhibit antifungal activity, including chitin degradation (Tran et al. 2024). Furthermore, the *P. polymyxa* J2-4 strain was recently shown to significantly promote cucumber growth under field conditions (Shi et al. 2024).

*Paenibacillus* spp. have also demonstrated effectiveness in protecting plants against phytopathogens through diverse biocontrol strategies. These mechanisms have proven effective against a broad spectrum of agriculturally relevant pathogens, including phytopathogenic fungi (Samain et al. 2017; Tran et al. 2024), pathogenic bacteria (Taheri et al. 2022a, b), and plant-parasitic nematodes (Singh and Wesemael 2022a, b; Shi et al. 2024). The production of bioactive secondary metabolites is a hallmark of *Paenibacillus* strains. Notably, *P. polymyxa* produces fusaricidin—a non-cationic cyclic lipopeptide with potent antifungal activity—and polymyxin, a cationic cyclic lipopeptide that is highly effective against Gram-negative bacteria (Raza et al. 2008).

*Paenibacillus* strains have shown strong potential for the biological control of root-knot nematodes belonging to the genus *Meloidogyne*. A prominent example is the *P. polymyxa* LMG27872 strain, which exhibited antagonistic activity against *Meloidogyne incognita* in both in vitro and in vivo experiments, while also promoting tomato growth (Singh and Wesemael 2022a, b). Beneficial and mutualistic rhizobacteria such as *Paenibacillus* spp., which colonize plant roots, can also trigger induced systemic resistance (ISR)—a defense mechanism that enhances plant immunity in distal tissues, providing broad-spectrum protection against pathogens and pests (Grady et al. 2016). *Paenibacillus* species have been shown to induce ISR against a wide array of pathogens, including bacteria (Lee et al. 2012; Phi et al. 2010a, 2010b), fungi (Gkizi et al. 2016), nematodes (Khan and Kim 2012), and viruses (Kumar et al. 2016).

To date, numerous *Paenibacillus* genomes have been sequenced, offering critical insights into the genetic basis of their functional diversity (Huang et al. 2020). Despite these advances, a systematic and comprehensive comparative genomic analysis is still needed to elucidate the genetic determinants underlying plant growth promotion and biocontrol capabilities. Such studies are essential for understanding the prevalence and distribution of these traits, as well as for elucidating the ecological roles of *Paenibacillus* species particularly within the rhizosphere and in association with plant hosts. In this context, we conducted a comparative genomic analysis of 428 *Paenibacillus* genomes to investigate the distribution and phylogenetic patterns of plant growth-promoting and biocontrol-related traits. Subsequently, a subset of 97 complete genomes was selected for in-depth functional analyses, enabling the identification of promising candidate species with potential as plant growth promoters and antagonists of phytopathogens. Additionally, we assessed potential risks, including the presence of genes associated with antimicrobial resistance and virulence factors.

## Materials and methods

### Genomic data collection and *Paenibacillus* strain distribution analysis

As of August 2024, approximately 1,500 *Paenibacillus* genome sequences had been deposited in GenBank. From these, 1,345 genomes from the RefSeq database were downloaded for quality assessment using CheckM v1.0.13 (Parks et al. 2015) and BUSCO (Seppey et al. 2019). Genomes meeting the criteria of > 95% completeness and < 0.5% contamination were retained, resulting in a final dataset of 428 high-quality genomes. Among these, 48 genomes belonged to *P. polymyxa*, while the remaining 380 represented other *Paenibacillus* species. Metadata regarding the geographic and environmental origins of each strain were retrieved from NCBI and are summarized in (Supplementary Table S1).

### Phylogenetic analysis

A phylogenetic reconstruction of the *Paenibacillus* core genome was performed using the 428 selected genomes. CheckM v1.0.13 (Parks et al. 2015) was employed to extract 43 single-copy universal marker genes using the tree_ga function. These marker protein sequences were concatenated and used for phylogenetic inference via the maximum likelihood method in IQ-TREE v1.6.11 (Minh et al. 2020), applying the best-fit model "LG + I + G4". Phylogenetic tree construction was conducted with the parameters –m TEST –bb 1000 to ensure robustness and statistical support. The resulting tree was visualized and annotated using the Interactive Tree of Life (iTOL) tool (Letunic and Bork 2019).

### Identification of plant growth-promoting traits (PGPTs) and biocontrol-associated genes

For functional analyses, only complete genomes were selected, which were annotated using Bakta (Schwengers et al. 2021). Additional details on these genomes are provided in Supplementary Table S2. The comprehensive PGPg_finder pipeline (Pellegrinetti et al. 2024) was used, combining direct sequence annotation with de novo assembly methods to accurately detect the presence of PGPTs in the genomes, encompassing categories of direct and indirect plant growth promotion. The tool cross-references the analyzed sequences with the PlaBAse database (Patz et al. 2021), enabling the identification and quantification of PGPTs in the evaluated genomic datasets. A total of 59 PGPT-related genes, annotated using PgPg_finder and associated with processes such as biofertilization and phytohormone production, were used as indicators of plant-interactive potential (Supplementary Table S3). The frequency of detected genes was transformed using the natural logarithm [ln(x)] and visualized as a heatmap using the online tool ClustVis v1.0 (Metsalu and Vilo 2015). Biosynthetic gene clusters (BGCs) were identified using antiSMASH v7.0 (Blin et al. 2023a, b), while carbohydrate-active enzymes (CAZymes) were annotated through the dbCAN3 pipeline (Zheng et al. 2023), using default detection thresholds.

### Pan-genome analysis and functional categorization (COGs)

A subset of 97 *Paenibacillus* genomes was selected for pan-genome analysis based on genome completeness and assembly quality. Only complete or near-complete genomes with low levels of fragmentation were included in order to minimize annotation artifacts and ensure reliable gene presence/absence inference, which is essential for comparative genomic and functional analyses. This quality-based selection aimed to increase the robustness of the pan-genome analysis. The genomes were annotated using Bakta (Schwengers et al. 2021) and the resulting protein FASTA files were input into the Bacterial Pan Genome Analysis (BPGA) v1.3 pipeline (Chaudhari et al. 2016). This pipeline facilitated clustering into core, accessory, and unique gene families and functional classification according to the Clusters of Orthologous Groups (COG) database. Pan-genome openness was assessed using Heaps’ law, described by the equation *n* = *um·x^b* (Tettelin et al. 2008), where *x* is the number of genomes and *um* and *b* are curve-fitting parameters. A value of *0* < *b* < *1* indicates an open pan-genome, while *b* < *0* suggests a closed one.

### Detection of mobile genetic elements (MGEs)

To investigate mobile genetic elements (MGEs), chromosomal sequences from complete genomes were obtained from NCBI and analyzed using ICEscreen v1.3.2 (Lao et al. 2022) to detect Integrative and Conjugative Elements (ICEs) and Integrative and Mobilizable Elements (IMEs). Only elements with associated signature proteins were considered. ICEs and IMEs were classified into four categories: complete ICEs (possessing recombination, conjugation, stability, integration, and excision modules), complete IMEs (with integration, excision, recombination, and mobilization modules), partial ICEs (with missing components), and other elements (degenerate or unclassified). Transposable elements were identified through BLASTn searches against the TnCentral, INTEGRALL, and ISFinder databases, using thresholds of > 50% coverage and > 70% identity (Ross et al. 2021). Plasmid content was obtained directly from complete genome entries in NCBI. In addition, genes associated with plant growth promotion (PGPTs) and biocontrol, identified using the antiSMASH 7.0, PGPT-Pred, and PGPg_finder tools, were correlated with the geographical locations of isolation and environmental types to assess the environmental influence on the abundance of PGPT genes and secondary metabolites. All identified elements were compared with each other by BLASTn using the Geneious software (https://www.geneious.com) to detect possible gene transfer events. ICEs and IMEs were also compared with the ICEberg 3.0 database (https://tool2-mml.sjtu.edu.cn/ICEberg3/ICEfinder.php) (Wang et al. 2024), being considered similar when they showed coverage greater than 50% and identity above 70%.

### Detection of antibiotic resistance genes and virulence factors

To assess the potential risks associated with antimicrobial resistance and virulence, we conducted a comprehensive screening of antibiotic resistance genes (ARGs) and virulence factors (VFs) across all *Paenibacillus* genomes analyzed. Gene sequence data were queried against multiple curated reference databases. Specifically, ARGs were identified using the Comprehensive Antibiotic Resistance Database (CARD; Alcock et al. 2020), ResFinder (Zankari et al. 2012), MEGARes (Lakin et al. 2017), and ARGANOT (Gupta et al. 2014). Virulence-associated genes were detected using the Virulence Factor Database (VFDB) implemented via the ABRicate tool (https://github.com/tseemann/abricate). Default parameters were used, with a minimum identity cutoff of 80% and minimum coverage threshold of 70%

### Data availability

All genomes used in this study are publicly available through the GenBank database (https://www.ncbi.nlm.nih.gov/genCiteESM.bank/.). Supplementary datasets generated and analyzed during this research are provided as supplementary materials accompanying this article.

## Results

### *Paenibacillus* strains are widely distributed in a variety of environments, demonstrating high ecological versatility

To investigate the global distribution and ecological range of *Paenibacillus* species, we compiled a comprehensive dataset from NCBI comprising the 428 high-quality genomes analyzed in this study (Supplementary Table S1). Environmental metadata was used to infer the origin of each strain, revealing considerable heterogeneity in isolation sources. Genomes were derived from diverse habitats, including marine sediments, non-agricultural soils, rhizospheric soils, food ingredients, and seeds, among others. Notably, 13.12% of the genomes were isolated from rhizospheric soils, reinforcing the ecological association of certain *Paenibacillus* strains with plant hosts.

**Fig. 1:**
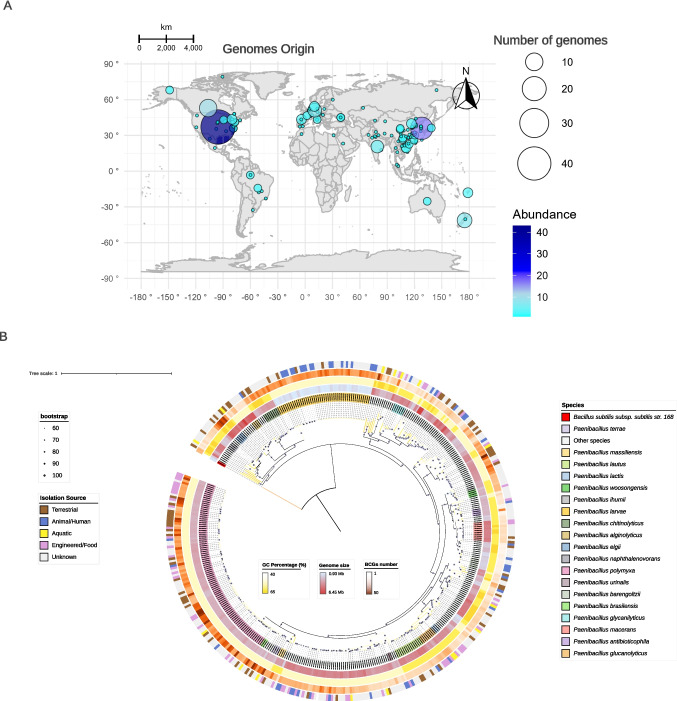
Global distribution and phylogeny of the *Paenibacillus*. (**A**) The global distribution of *Paenibacillus* described in this study is represented on a map generated using the ‘ggplot 2’ package in R, with abundance highlighted by circles of varying sizes (Figure A). (**B**) Phylogenetic tree constructed using maximum likelihood method with IQ-TREE v.1.6.11, based on 428 *Paenibacillus* genomes. The analysis utilized 43 single-copy marker genes, identified using CheckM, and was performed under the LG + I + G4 rate heterogeneity model with maximum of 1,000 bootstrap replicates. Bootstrap values are represented by black circles. Each color in the phylogenetic tree corresponds to a clade representing distinct species, as indicated in the legend, including the outgroup *Bacillus subtilis*. The tree is scaled, with branch lengths in the same units as the evolutionary distances used to infer the phylogenetic tree

Geographical analysis showed that the genomes were distributed across multiple continents (Fig. 1A). Genome size among the 428 strains varied widely, ranging from 0.93 Mb to 6.45 Mb, while G + C content spanned from 40.0% to 65.0%. The number of biosynthetic gene clusters (BGCs) identified per genome ranged from 1 to 50. The *P. polymyxa* clade stood out for harboring a notably high number of BGCs, suggesting strong secondary metabolic potential within this group.

To explore evolutionary relationships within the genus, we constructed a robust phylogenetic tree based on the concatenated alignment of 43 universally conserved single-copy marker genes (Fig. 1B). The tree, derived from 428 publicly available RefSeq genomes, exhibited strong bootstrap support for most nodes (> 90%), indicating high phylogenetic reliability. Our analysis resolved 28 well-defined branches, each representing a distinct taxonomic lineage, thereby highlighting the extensive species diversity encompassed within the genus.

Most of the genomes were found to cluster within the *P. polymyxa* clade, the type species of the genus (Trüper, 2005), reflecting both its ecological prominence and its appeal as a model organism for functional studies. The increasing scientific interest in this species, coupled with advancements and accessibility in sequencing technologies, has led to a notable rise in genomic submissions to public repositories in recent years (Wallner et al. 2024). Other highly represented clades included *P. larvae*, *P. lautus*, *P. macerans*, and *P. chitinolyticus*, each contributing to the overall genomic and functional diversity observed across the dataset.

### *Paenibacillus* genomes exhibit diverse genetic repertoires for plant growth-promoting traits (PGPTs)

To identify genes associated with plant growth-promoting traits (PGPTs), we analyzed 97 complete *Paenibacillus* genomes by screening their predicted protein sequences. The PGPTs were classified into broad functional categories involving direct and indirect mechanisms of plant growth promotion. Among the indirect categories, genes related to the colonization of plant-derived substrates and abiotic stress mitigation were particularly prominent. In the direct categories, the most notable mechanisms included iron acquisition, plant vitamin biosynthesis, and phosphate solubilization (Supplementary Fig. 1). On average, the genomes contained approximately 249 genes related to biofertilization, around 190 genes associated with phytohormone production, 97 genes involved in bioremediation, and approximately 322 genes linked to competitive exclusion. These categories represent the major functional groups of plant growth-promoting traits (PGPTs) identified across the analyzed genomes.

Species such as *P. mucilaginosus* (987 genes), *P. thiaminolyticus* (920 genes), and *P. borealis* (919 genes) exhibited the highest total numbers of PGPT-related genes, particularly within the biofertilization category, surpassing even *P. polymyxa* (850 genes). These findings suggest that these species may play a more significant role in promoting plant growth.

Analysis revealed substantial variation in the frequency and distribution of PGPT-related genes across the analyzed genomes, highlighting the functional diversity and differential plant-associative potential among *Paenibacillus* species. Strains isolated from soil environments exhibited the highest absolute numbers of genes related to plant growth promotion compared to isolates from other environments, totaling 5,522 genes associated with biofertilization, 2,288 genes related to bioremediation, 4,164 genes linked to phytohormone production, and 7,057 genes related to competitive exclusion. However, when the average number of PGPT-related genes per isolate was calculated for each environment to correct for sampling size bias, isolates from plant-associated environments (900) and the rhizosphere (874) showed higher averages than those from soil (865).

**Fig. 2:**
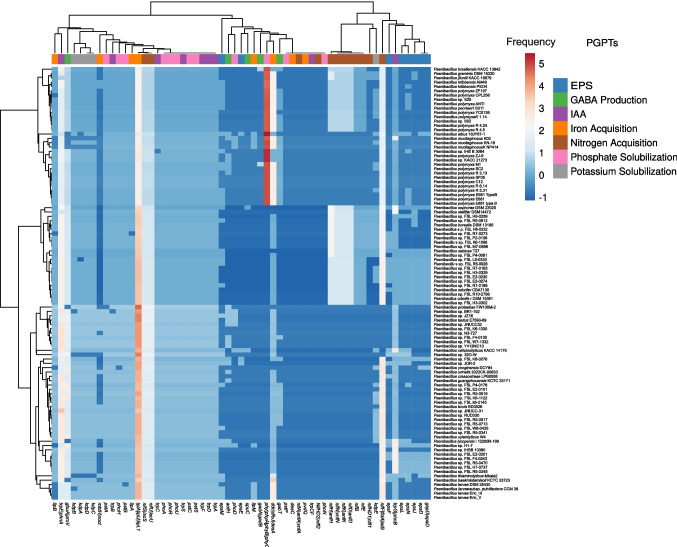
A heatmap describing the distribution of genes associated with plant growth-promoting traits (PGPTs) that directly contribute to plant growth. The abundance values were log-transformed using ln(x). Row centering and unit variance scaling were applied to improve visualization. Missing values were estimated using imputation techniques. Rows and columns were clustered using correlation distance and average linkage methods

As illustrated in Fig. 2, genes related to phosphate solubilization—*phyA* (phytase A), *phyB* (phytase B), and *phyC* (phytase C)—were among the most frequently detected, occurring up to 14 times in individual genomes. These genes were predominantly found in *P. polymyxa*, *P. kribbensis*, *P. brasilensis*, and *P. graminis*. Additional phosphate metabolism genes, including *phoA* (alkaline phosphatase), *phoD* (alkaline phosphatase D), *phoH* (phosphate starvation-inducible protein), and *phoR* (phosphate regulon sensor kinase), were less abundant, although phoA showed a consistent presence across all analyzed genomes.

Siderophore-associated genes, particularly *dfoJ* (desferrioxamine biosynthesis protein) and *desA* (desferrioxamine biosynthesis enzyme), were detected in all genomes, with frequencies ranging from two to eight copies per genome, suggesting a conserved genetic potential for iron acquisition. Genes associated with indole-3-acetic acid (IAA) biosynthesis — including *trpA* (tryptophan synthase alpha subunit), *trpB* (tryptophan synthase beta subunit), *trpC* (indole-3-glycerol phosphate synthase), *trpD* (anthranilate phosphoribosyltransferase), *trpE* (anthranilate synthase component I), *iaaM* (tryptophan monooxygenase), *iaaH* (indole-3-acetamide hydrolase), *ipdC* (indole-3-pyruvate decarboxylase), and *ppdC* (phenylpyruvate decarboxylase) — were detected across several *Paenibacillus* genomes (Supplementary Table S3). These genes are mainly associated with tryptophan-dependent auxin biosynthesis pathways, particularly the indole-3-pyruvate (IPyA) pathway, which is widely reported in plant-associated bacteria. Although auxin-related genes were broadly distributed, differences in gene composition and copy number were observed among species, indicating variability in the metabolic potential for auxin biosynthesis within the genus (Supplementary Table S3). The presence of these genes suggests a genetic potential for auxin biosynthesis within the genus, although the completeness of individual pathways requires further investigation.

Regarding γ-aminobutyric acid (GABA) production, the genes *gbuA* (GABA permease subunit) and *proV* (glycine betaine/proline ABC transporter substrate-binding protein) were widely distributed, appearing in most genomes with frequencies between four and six copies. Genes associated with potassium solubilization—*kdpA* (potassium-transporting ATPase subunit A), *kdpB* (subunit B), *kdpC* (subunit C), and *kdpD* (sensor histidine kinase)—were present in most genomes at a frequency of approximately two copies each. More than 20 *Paenibacillus* species are known to fix atmospheric nitrogen, and the nitrogen fixation (*nif*) gene cluster remains highly conserved among these nitrogen-fixing strains (Xie et al. 2014). In our dataset, nine key genes involved in nitrogen fixation—*nifA* (transcriptional regulator), *nifB* (FeMo-cofactor biosynthesis protein), *nifD* (nitrogenase alpha subunit), *nifK* (nitrogenase beta subunit), *nifS* (cysteine desulfurase), *nifU* (Fe–S cluster scaffold protein), *nifH* (nitrogenase reductase), *nifF* (ferredoxin), and *nifN* (FeMo-cofactor biosynthesis protein)—were identified. Among these, *nifS* and *nifU* were ubiquitous across all analyzed genomes, whereas *nifK*, *nifD*, and *nifH* were present in approximately 38% of the genomes, generally with lower copy numbers (two to six). The species with the highest representation of nitrogen fixation-related genes included *P. polymyxa*, *P. jilunlii*, *P. sabinae*, and *P. brasiliensis*. Finally, genes related to exopolysaccharide (EPS) biosynthesis—*epsL* (glycosyltransferase), *epsJ* (EPS biosynthesis protein), *epsE* (priming glycosyltransferase), and *epsM* (EPS export protein)—were detected at low frequencies, with most genomes containing no more than two copies. Despite their limited abundance, the presence of these genes suggests a potential for biofilm formation and enhanced root colonization in certain strains.

### The *Paenibacillus* pan-genome is open and functionally diverse across strains

**Fig. 3:**
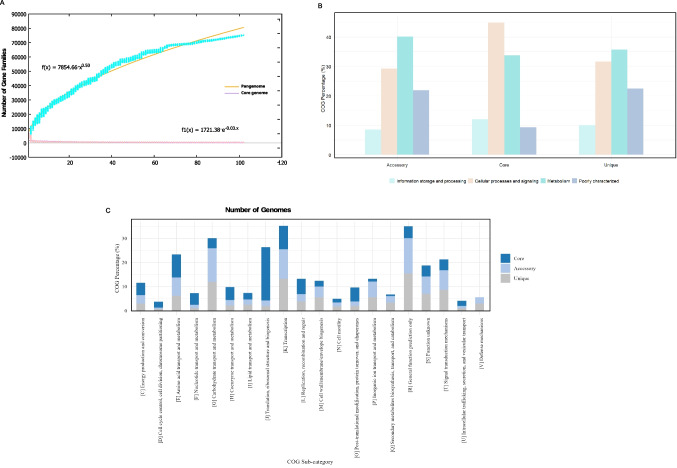
Graphic representation of the pan-genome and core-genome of 97 complete *Paenibacillus* genomes. (**A**) The plot shows that the equations fit the total and core genome families, as well as how the number of gene families increases and decreases in the pan-genome and core-genomes as more genomes are added. (**B**) Displays the functional proportions of core, accessory, and unique genes in the COG categories. (**C**) Distribution of COG subcategories

The pan-genome analysis of *Paenibacillus* strains revealed a total of over 80,000 gene families, with an average of 418 core genes, 4,631 accessory genes, and 355 unique genes per genome. Core genes accounted for approximately 7.73% of each genome, while accessory and unique genes represented, on average, 85.7% and 6.57%, respectively. According to Heaps’ Law (*b* = 0.503), the *Paenibacillus* pan-genome remains open and expanding, reflecting high genomic plasticity and the continuous acquisition of new genes (Fig. 3A). Notably, species such as *P. thiaminolyticus*, *P. cellulosilyticus*, and *P. baekrokdamisoli* exhibited the highest proportions of unique, species-specific genes, suggesting specialized ecological roles.

Functional classification based on Clusters of Orthologous Groups (COG) demonstrated distinct patterns among gene categories (Fig. 3B). The core genome was primarily enriched in genes associated with cellular processes and signaling (44.86%), particularly within class J (22.28%; translation, ribosomal structure and biogenesis), class E (9.63%; amino acid transport and metabolism), and class C (5.11%; energy production and conversion). In contrast, these same functional classes were proportionally reduced in the accessory (J: 2.31%, E: 7.54%, C: 3.49%) and unique genomes (J: 1.89%, E: 6.23%, C: 2.96%). Meanwhile, the accessory and unique gene fractions were strongly enriched in functions associated with environmental adaptation and defense, including class K (12.24% and 13.28%; transcription), class G (13.98% and 11.95%; carbohydrate transport and metabolism), class T (8.10% and 8.71%; signal transduction), class Q (2.77% and 3.35%; secondary metabolite biosynthesis, transport, and catabolism), and class V (2.63% and 2.95%; defense mechanisms) (Fig. 3C). Collectively, these findings indicate that while the core genome retains genes essential for fundamental cellular maintenance, the accessory and unique genomes provide the functional versatility necessary for environmental resilience, host interaction, and niche specialization across diverse *Paenibacillus* strains.

### The broad variability of biosynthetic genes in *Paenibacillus* lineages suggests the existence of a shared metabolic core

Analyses performed with AntiSMASH identified numerous secondary metabolite clusters across the *Paenibacillus* genomes. A total of 1,064 Biosynthetic Gene Clusters (BGCs) were identified and distributed into 163 different types within our dataset. The most frequent BGC was the NRP (Non-Ribosomal Peptides), with 130 occurrences, representing 100% of the maximum frequency. Other prominent clusters included RiPP (Ribosomally Synthesized and Post-Translationally Modified Peptides) with 85 occurrences (65.38%) and NRP + Polyketide with 55 occurrences (42.31%). Less frequent clusters, such as NRP: Glycopeptide and Polyketide: Iterative type I polyketide + Saccharide, were observed only once each, representing 0.77% of the total. In addition, a total of 128 unique metabolite types were identified, reflecting the significant diversity of compounds produced by the genus *Paenibacillus*. The strains with the largest biosynthetic regions were *P. larvae* subsp. *larvae* strain Eric_III (21 regions) and *P. polymyxa* strain R 3.13 (20 regions) (Supplementary Table S5).

**Fig. 4:**
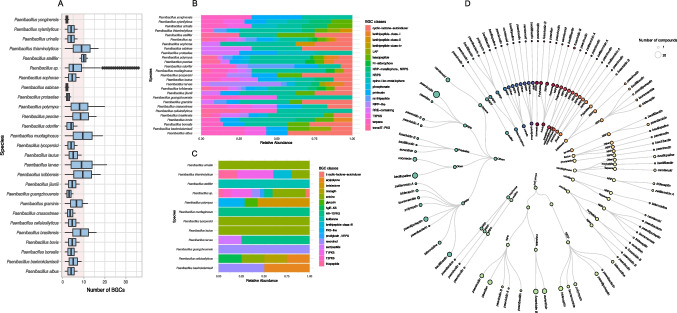
Abundance and diversity of biosynthetic gene clusters (BGCs) and their associated secondary metabolites across species of the genus *Paenibacillus*. **A**. Boxplot showing the distribution of the number of BGCs across *Paenibacillus* species. The red highlight indicates the average of 9.5 BGCs per genome. **B**. Relative abundance of core BGC classes across different *Paenibacillus* species. **C**. Relative abundance of exclusive BGC classes across different *Paenibacillus* species. Circle size represents the normalized number of a specific compound. **D**. Distribution of bioactive secondary metabolite compounds within *Paenibacillus* species. Additional details are available at Supplementary Table S5

A comparative analysis of the number of BGCs identified across different *Paenibacillus* species revealed substantial variability, with an average of 9.5 BGCs per genome (Fig. 4A). The average number of BGCs per species varied considerably, with *P. larvae*, *P. polymyxa*, and *P. mucilaginosus* exhibiting the highest averages (20, 18, and 17, respectively), while species such as *P. sabinae* and *P. yonginensis* showed lower numbers. Notably, *Paenibacillus sp.* exhibited a broad dispersion in the number of BGCs (Fig. 4A), indicating significant genomic diversity within this unidentified group. This pattern may reflect the inclusion of heterogeneous genomes or differences in environmental conditions at the time of isolation.

The presence of conserved BGCs across *Paenibacillus* species suggests the existence of a shared metabolic core (Fig. 4B). Classes such as transAT − PKS, T3PKS, proteusin, NRPS, lanthipeptide-class-I, lanthipeptide-class-II, and terpenes were widely distributed among species, representing a significant fraction of the BGCs in nearly all the strains analyzed. In contrast, several species exhibited biosynthetic classes that were either exclusive or rarely shared (Fig. 4C). These specific classes are indicative of metabolic specializations and may be linked to ecological adaptations or unique environmental interactions. Species such as *P. polymyxa* and *P. mucilaginosus* featured classes like glycoin and betalactone, which were not widely represented in other species. *P. baekrokdamisoli* and *P. guangzhouensis* showed high proportions of resorcinol (Fig. 4C). Other classes, such as thiopetide, prodigiosin, ectoine, and crocagin, were confined to a small set of species, underscoring their functional exclusivity.

Finally, we gained insights into the distribution of key bioactive compounds within the genus (Fig. 4D). Analysis of our dataset revealed that a considerable portion of well-characterized secondary metabolite clusters with potential biocontrol activity exhibited similarity greater than 70%. The most abundant BGCs included Bacillopaline, Tridecaptin, Fusaricidin B, Bacillibactin, Paeninodin, and Polymyxin, with Bacillopaline (24), Tridecaptin (22), and Fusaricidin B (22) being the most prominent. In total, 186 clusters with similarity > 70% were identified. The results indicate that *P. polymyxa* strains harbor the highest number of well-characterized BGCs for biological control, with an average of seven clusters per genome. In addition to *P. polymyxa*, other strains, such as *Paenibacillus thiaminolyticus* Mbale2, *Paenibacillus cellulosilyticus*, and *P. cellulosilyticus* KACC 14175, also presented a considerable number of BGCs, including Polymyxin, Ectoine, Paeninodin, Fusaricidin B, and Tridecaptin. Furthermore, our findings suggest that species such as *Paenibacillus brasilensis*, *Paenibacillus peoriae*, and *Paenibacillus kribbensis* have potential for biocontrol of phytopathogens, as highly characterized BGCs, including Fusaricidin B, Bacillopaline, and Tridecaptin, were found with 100% similarity to previously described clusters.

### Exploring the potential of *Paenibacillus* in the degradation of plant-based polymers

Carbohydrate-active enzymes (CAZymes) play a fundamental role in plant growth and defense, particularly in the agricultural context, where they contribute to the synthesis, remodeling, and degradation of structurally diverse carbohydrates. Through genome-wide analysis of complete *Paenibacillus* genomes, we identified 30 distinct CAZyme families (Supplementary Table S6), with glycoside hydrolases (GHs) emerging as the most dominant group, while polysaccharide lyases (PLs) were comparatively less represented.

**Fig. 5:**
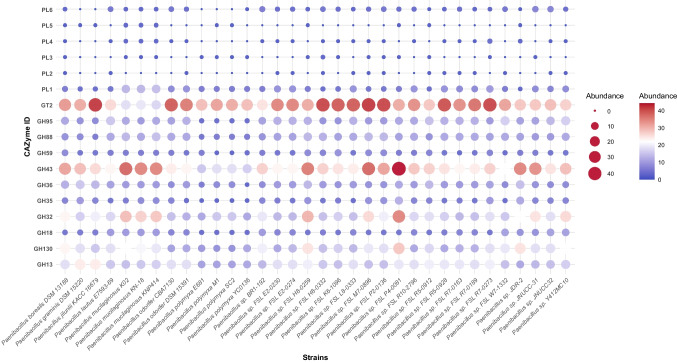
Bubble plot illustrating the abundance of genes involved in carbohydrate degradation across *Paenibacillus* strains. The abbreviation “GH” refers to glycoside hydrolases. The x-axis represents the genes, while the y-axis displays *Paenibacillus strains*

Among all identified families, five were particularly abundant: GT2 (18.75%), GH43 (12.90%), GH32 (9.00%), GH13 (8.87%), and GH130 (7.50%), collectively accounting for 1,172 to 2,935 hits across the dataset (Fig. 5). These enzymes are functionally associated with the breakdown of a wide range of plant-derived polysaccharides, including cellulose, hemicellulose, arabinoxylan, inulin, sucrose, starch, glycogen, and mannose-rich oligosaccharides. Notably, species such as *P. borealis*, *P. mucilaginosus*, *P. jilunlii*, and *P. polymyxa* exhibited particularly high levels of these CAZyme families, suggesting a strong capacity for complex carbohydrate degradation (Fig. 5).

**Fig. 6:**
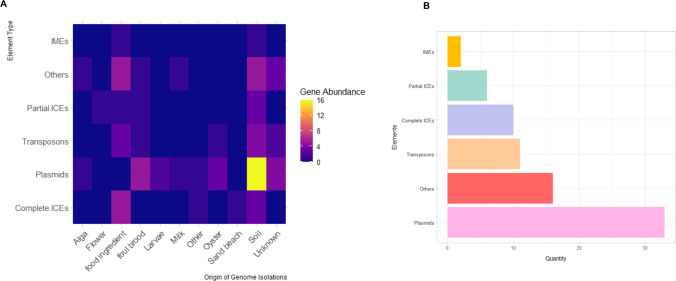
Heatmap illustrating the abundance of mobile genetic elements in *Paenibacillus* genomes (**A**). The figure correlates the number of plant growth promotion genes present in each element (Y-axis) and the isolation source of the strains (X-axis). The total quantity of each element identified in the strains (**B**)

In addition, the GH88 and GH95 families were especially enriched in these species, indicating their potential involvement in the degradation of uronic acid components of fucose-rich glycoproteins. These findings underscore the metabolic versatility of *Paenibacillus* and its potential relevance in biomass turnover and rhizosphere competence. Of particular interest was the widespread presence of GH18 family enzymes, which include bacterial chitinases—key players in biocontrol against fungal pathogens. Genes such as *chiA*, *chiB*, *chiC*, and *chiD* were detected in nearly all genomes, with *P. guangzhouensis* and *P. mucilaginosus* standing out due to their high content of GH18 enzymes, averaging 12 and 9 copies, respectively. These results strongly support the hypothesis that *Paenibacillus* chitinases contribute to both direct antagonism of phytopathogens and indirect plant growth promotion.

### Mobile genetic elements in *Paenibacillus* genomes

Mobile genetic elements (MGEs) are major drivers of genomic plasticity, horizontal gene transfer, and microbial adaptation, making their analysis essential to understanding the dissemination of functional traits such as plant growth promotion and biocontrol potential in microbial populations. In this study, we investigated MGEs in *Paenibacillus* genomes to assess their diversity, genomic context, and potential contribution to ecological functions.

Using ICEscreen, we identified a total of 34 integrative elements, including nine complete integrative and conjugative elements (ICEs), seven partial ICEs, two complete integrative and mobilizable elements (IMEs), and sixteen degenerated or unclassified elements (Fig. 6). These elements ranged in size from 3,857 bp to 141,122 bp, with GC content varying from 36.0% to 54.4%. Although ICEs and IMEs exhibited limited representation of plant growth-promoting traits (PGPTs) and biocontrol-related genes, they frequently contained *lplA* and, in some cases, *nod* and *pho* genes (Supplementary Table S7), with a higher occurrence of genes in the categories competitive exclusion (23) and biofertilization (23), suggesting a potential, albeit modest, role in the dissemination of functional traits. None of the ICEs or IMEs showed matches in ICEBerg 3.0 and were therefore considered novel.

In parallel, twelve sequences related to transposable elements (transposons and insertion sequences) were identified, ranging from 353 bp to 1,770 bp and with GC content between 35.8% and 53.8%. These elements were predominantly recovered from soil and aquatic environments and showed no association with PGPTs or biocontrol genes.

In contrast, plasmids demonstrated greater functional relevance. A total of 35 plasmids were identified, showing remarkable size variation—from 973 bp to 2,697,188 bp—and GC content between 37.1% and 52.0%. These plasmids harbored a richer set of plant growth-promoting genes, comprising 44 genes distributed across specific categories such as nodulation and IAA production, and totaling 996 plant growth-promoting genes overall. Notably, one plasmid from Paenibacillus cellulosilyticus contained 137 bio-fertilization genes and 91 phytohormone-related genes (Supplementary Table S8). Furthermore, all plasmids included 15 biosynthetic gene clusters (BGCs) associated with the production of antimicrobial compounds such as paenilipoheptin, thermoactinoamide A, and bacillibactin.

Most functionally enriched plasmids were derived from strains isolated from soil (*n* = 33), feed ingredients (*n* = 13), and unknown sources (*n* = 9), underscoring the environmental relevance of MGEs in shaping the functional repertoire of P. populati. Additionally, four partial elements were found to be similar to each other and detected in different locations, although mostly recovered from the same type of environment (two from soil and two of unknown origin). This finding highlights the importance of MGEs as vectors for gene exchange, contributing to microbial community maintenance and ecological interactions within the genus *Paenibacillus* and its associated environments.

### Pathogenicity and antibiotic resistance in *Paenibacillus* genomes

No virulence-associated genes were identified across the 97 *Paenibacillus* genomes analyzed. In contrast, a limited yet phylogenetically diverse repertoire of antibiotic resistance genes (ARGs) was detected. The most prevalent gene was *rphB*, associated with rifampicin resistance, present in 47 genomes (46.1%). This was followed by *tetA* and *tetB*, related to tetracycline resistance, detected in 14 genomes (13.7%). Additional ARGs were identified at lower frequencies and spanned resistance mechanisms to aminoglycosides (*aadE-Pp*), macrolides (*mphI*), lincosamides, phenicols, pleuromutilins (*cipA, lsaB, TaeA*), and glycopeptides (*vanF/vanA*) (Supplementary Table S9).

The strains with the highest number of ARGs were *P. lautus*, *Paenibacillus* sp. FSL W7-1332, and *Paenibacillus* sp. JZ16, each carrying up to 12 distinct resistance genes. These strains exhibited multidrug resistance profiles, including genes conferring resistance to tetracyclines, macrolides, aminoglycosides, lincosamides, and pleuromutilins.This finding suggests that, despite the generally low prevalence of ARGs across the genus, certain strains may serve as reservoirs of resistance genes. These results underscore the importance of monitoring gene mobility and potential environmental dissemination. For detailed annotations and gene distribution, refer to (Supplementary Table S9).

## Discussion

### Deciphering the biocontrol potential in *Paenibacillus* strains

Recent studies have demonstrated that *Paenibacillus* strains play a key role in the suppression of phytopathogens (Kim et al. 2019; Singh and Wesemael 2022a, b; Taheri et al. 2022a, b). Most of the current research has focused on *P. polymyxa*, a species that has attracted considerable attention due to its extensive potential across various industrial and agricultural sectors (Huang et al. 2024). However, there remains a clear need to investigate other species within the genus, particularly about their applications in sustainable agriculture.

To our knowledge, no systematic and comprehensive comparative genomic analysis has been conducted to investigate intrinsic features related to biocontrol and plant growth promotion across the *Paenibacillus* genus. In this study, we addressed this gap by selecting high-quality genomes to explore the functional potential of diverse *Paenibacillus* species. We first mapped the carbohydrate degradation profile of *Paenibacillus*. Our results revealed an extensive enzymatic repertoire among strains, with some encoding over 260 CAZymes, particularly in species also enriched in PGPT genes. The GH family (Glycoside Hydrolases), the largest and most studied group of CAZymes (Wardman et al. 2022), was the most dominant, with GH18 (chitinases) being ubiquitous across all strains. Chitinases hydrolyze β−1,4 glycosidic bonds in chitin and chitosan oligosaccharides, and play a key role in degrading fungal cell walls, insect exoskeletons, and nematode eggshells (Chen et al. 2020a, 2020b, 2020c; Moussian 2009).

The production of chitinases has been widely documented among *Paenibacillus* representatives (Du et al. 2021a, b; Veliz et al. 2017a, 2017b; Zhang et al. 2021). For example, *P. elgii* strain HOA73 was shown to control Gray Mold in tomato with efficacy comparable to that of commercial fungicides (Kim et al. 2019). Our comparative genomic analysis revealed a high abundance of GH18 enzymes in *P. mucilaginosus*, consistent with previous studies reporting its chitinolytic activity in vitro (Doan et al. 2019). The results also indicated that most *Paenibacillus* strains possess genes from GT2 and GH43 families, involved in the degradation of cellulose and hemicellulose, reinforcing the genus's capacity to utilize a broad range of carbon sources and adapt to soil environments.

Our analysis identified 128 distinct classes of secondary metabolites within the *Paenibacillus* genomes, reflecting the remarkable biosynthetic diversity of this genus. The most frequently detected clusters included NRPS, RiPPs, and polyketide hybrids, many of which are associated with antimicrobial activity. Several known bioactive compounds produced by *Paenibacillus*, such as fusaricidin, pelgipeptin, polymyxin B, tridecaptin, and colistin, were identified in our dataset (Dobrzyński and Naziębło 2024). Additionally, ribosomally synthesized peptides like paenicidin B were also observed (Lebedeva et al. 2021).

Secondary metabolites are low-molecular-weight bioactive compounds that, although not essential for primary metabolism, confer significant ecological advantages (Dewick 2009). The genes responsible for their biosynthesis are typically organized into discrete genomic regions known as biosynthetic gene clusters (BGCs) (Blin et al. 2023a, 2023b). Among these, non-ribosomal peptides (NRPs) are particularly abundant and include numerous antimicrobial agents and immunomodulators. NRPS (Non-Ribosomal Peptide Synthetases), modular enzymes responsible for the biosynthesis of these compounds, are widely recognized for their pharmaceutical and agricultural applications (Iacovelli et al. 2021).

Notably, some species stood out due to their high number of BGCs. *P. mucilaginosus* (18 BGCs), *P. peoriae* (17), *P. polymyxa* (17), and *P. kribbensis* (17) were particularly enriched. *P. mucilaginosus*, known for promoting plant growth, has been shown to produce NRPS-derived antimicrobials capable of suppressing *Fusarium oxysporum* (Wang et al. 2023b). Although the role of *P. kribbensis* BGCs in plant interactions remains unclear, many of its lassopeptides are known to inhibit microbial competitors (Mukhopadhyay et al. 2004). *P. peoriae* has been reported to possess antimicrobial activity against *Pectobacterium brasiliense*, attributed to its secondary metabolites (Zhao et al. 2022). In another study, *P. peoriae* HJ-2 showed antifungal efficacy against *Fusarium concentricum* in both greenhouse and field experiments involving *Paris polyphylla*, partly due to the presence of genes encoding fusaricidin synthetase (Jiang et al. 2022). Similarly, *P. polymyxa* WLY78 demonstrated that fusaricidin can induce systemic resistance via the salicylic acid (SA) signaling pathway against cucumber Fusarium wilt (Li and Chen 2019).

Finally, *P. polymyxa* remains the most widely studied species, with NRPS-based BGCs consistently linked to antimicrobial compound production, phytopathogen inhibition, and plant growth promotion (Jeong et al. 2019). These results emphasize the significance of *Paenibacillus* as a reservoir of biotechnologically relevant secondary metabolites and identify key species with promising applications in sustainable agriculture.

### Main *Paenibacillus* species involved in plant interactions and their potential for agricultural applications

Most *Paenibacillus* species have been isolated from soil environments and are frequently found in the plant rhizosphere, suggesting that many members of this genus possess intrinsic capabilities for promoting plant growth. Our genomic analyses revealed a consistently high abundance of plant growth-promoting trait (PGPT) genes in various *P. polymyxa* strains, reinforcing the prominent status of this species. Widely acknowledged for its versatility and biotechnological relevance, *P. polymyxa* has been referred to as the "jack of all trades" by Langendries and Goormachtig (2021), owing to its multifaceted applications in both industry and agriculture.

Numerous studies corroborate the efficacy of *P. polymyxa* in promoting plant growth through a range of direct and indirect mechanisms. Direct contributions include the synthesis of phytohormones (Sun et al. 2022), efficient production of biofertilizers such as butanediol (Ju et al. 2023), exopolysaccharides (Liyaskina et al. 2021), and biological nitrogen fixation in crops such as cucumber (Li et al. 2022a, b). Additionally, the species can solubilize key nutrients like phosphorus and zinc (Ahmad et al. 2021).

Species such as *P. mucilaginosus*, *P. thiaminolyticus*, and *P. borealis* exhibited the largest sets of genes associated with plant growth-promoting traits, particularly those related to biofertilization, indicating a potentially more relevant role in supporting plant growth. Although *P. polymyxa* remains the most studied and widely applied species, our results highlight that other species, such as *P. peoriae* and *P. kribbensis*, display comparable functional potential — each harboring the same total number of PGPT-related genes as *P. polymyxa*. Both *P. peoriae* and *P. kribbensis* have been investigated for their biocontrol properties (Yurong et al. 2022), and *P. kribbensis* is noted for its capacity to produce a wide array of secondary metabolites with antibacterial activity (Li et al. 2024). However, their roles as plant growth-promoting rhizobacteria (PGPRs) remain largely unexplored.

Additional species—such as *P. jilunlii*, *P. sophorae*, and *P. brasilensis*—also exhibited elevated frequencies of PGPT-related genes. Notably, *P. brasilensis* is a nitrogen-fixing bacterium associated with maize (Von der Weid et al. 2002) and has been shown to delay senescence in tangerines (Chen et al. 2020a, b, c), emphasizing the functional diversity within the genus. On average, the genomes analyzed in this study encoded approximately 96 PGPT-related genes, suggesting that the capacity for plant growth promotion is broadly distributed across *Paenibacillus* species.

Emerging evidence from the literature further supports the agricultural potential of less-explored species. For instance, *Paenibacillus monticola* enhances white clover seedling growth (Li et al. 2022a, 2022b), *P. lentimorbus* mitigates nutrient deficiencies in maize (Singh et al. 2024), and *P. mucilaginosus*, when co-inoculated with other beneficial rhizobacteria, improves soybean agronomic performance (Xing et al. 2022). As emphasized by Dobrzyński and Naziębło (2024), there is a pressing need to expand the scope of research beyond *P. polymyxa* to uncover the hidden potential of other *Paenibacillus* species. Our findings support this view, indicating that a broader array of species within the genus may be effectively employed in both the biocontrol of phytopathogens and the direct promotion of plant growth in agricultural systems.

### Phosphate solubilization and iron acquisition: predominant mechanisms of *Paenibacillus* in plant growth promotion

Phosphorus and iron are essential micronutrients required for optimal plant development (Sharma et al. 2013; Sultana et al. 2021). Despite their abundance in the soil, these elements are often inaccessible to plants due to their chemical forms. Phosphorus is typically immobilized through complexation with metal ions or incorporated into organic matter, rendering it insoluble (Rawat et al. 2021). Similarly, under alkaline conditions, iron (Fe^3^⁺) tends to precipitate as insoluble oxyhydroxides (Grady et al. 2016). This limited bioavailability frequently necessitates the application of agrochemicals to supplement phosphate or to correct soil pH (Sharma et al. 2013).

In this context, the use of bioinoculants has emerged as a sustainable and cost-effective alternative for enhancing nutrient availability in agricultural systems (Elnahal et al. 2022; Singh and Kumar 2024). Numerous *Paenibacillus* species have been reported for their capacity to solubilize inorganic phosphate and facilitate iron uptake (Grady et al. 2016; Soni et al. 2021; Yuan et al. 2022; Wang et al. 2023a, b; Wendisch et al. 2023; Dobrzyński and Naziębło 2024). Phosphate solubilization in *Paenibacillus* is often attributed to the production of organic acids such as gluconic acid, while iron acquisition may involve siderophore secretion, organic acid exudation, or induction of plant genes related to iron mobilization (Grady et al. 2016).

Our genomic analyses indicate that phosphate solubilization in *Paenibacillus* is predominantly mediated by organic acid production, mainly through the PQQ-dependent glucose oxidation pathway, which leads to gluconic acid synthesis and medium acidification. Genes associated with this pathway, including *gcd* and multiple *pqq* genes (*pqqC*, *pqqD*, *pqqE*, and *pqqL*), as well as genes involved in gluconate and keto-gluconate metabolism (*gnl*, *gnd*, *gadh*, and *ghrB*), were detected at high frequency across the analyzed genomes, supporting a conserved mechanism for inorganic phosphate solubilization. In addition, genes encoding phosphatases and genes involved in polyphosphate metabolism (e.g., *ppk* and *ppx*) were identified with a more heterogeneous distribution, suggesting complementary and species-specific phosphate acquisition strategies within the genus. Consistent with these mechanistic insights, our analyses indicate that the genetic potential for phosphate solubilization and iron acquisition is widely distributed across the genus. Notably, *P. polymyxa*, *P. kribbensis*, *P. brasilensis*, and *P. graminis* harbour an extensive repertoire of genes associated with phosphate solubilization and iron acquisition. Among these, the genes *phy*, *phyA*, *phyB*, and *phyC* were particularly abundant and recurrent. Experimental validation has confirmed phosphate solubilization in *P. polymyxa* (Mohd Din et al. 2020), *P. kribbensis* (Zhang et al. 2013), and *P. brasilensis* (Arthurson et al. 2011). In contrast, iron acquisition has been extensively characterized only in *P. polymyxa* (Zhou et al. 2016; Grady et al. 2016), indicating a knowledge gap regarding the expression of this trait in other *Paenibacillus* species.

The potential of *Paenibacillus* spp. for plant growth promotion, particularly through these nutrient acquisition strategies, is well documented (Grady et al. 2016). For example, *P. polymyxa* has demonstrated beneficial effects in several agronomically important crops, including rice (Abdallah et al. 2019), maize (Mohd Din et al. 2020), pepper (Phi et al. 2010a, 2010b), tobacco (Liu et al. 2020), and wheat (Li et al. 2023). However, despite the genomic potential observed in *P. kribbensis*, *P. brasilensis*, and *P. graminis*, their functional capacities remain underexplored.

Further in vitro and in vivo studies are necessary to assess the extent to which these lesser-studied species can solubilize phosphate and mobilize iron under field-relevant conditions. Such efforts could lead to the identification of novel, effective candidates for the development of next generation biofertilizers, contributing to more sustainable agricultural practices.

### Key characteristics that make *Paenibacillus* a promising genus for biotechnological applications

Species of the genus *Paenibacillus* have garnered growing interest over the past decades, primarily due to their biotechnological potential (Bloemberg and Lugtenberg 2001). Several intrinsic features support their candidacy as versatile tools for agricultural and industrial applications. One such characteristic is their motility via flagella, which enables *Paenibacillus* strains to detect and respond to root exudates and mucilage components, facilitating chemotactic movement and efficient root colonization. Another crucial trait is their ability to form endospores, which enhances their resilience to environmental stresses and improves their viability and shelf life in bioformulations (Dobrzyński and Naziębło 2024). This spore-forming capacity makes them highly suitable for large-scale applications in diverse and fluctuating field conditions.

In addition to traits directly associated with plant growth promotion, functional annotation based on the PGPG_finder pipeline revealed a consistent set of indirect effects related to environmental adaptation. Genes associated with root system colonization and the utilization of plant-derived substrates, such as *araR* and the *lplA–C* gene cluster, were widely distributed among the 97 analyzed genomes, suggesting an adaptive capacity related to the rhizosphere. This pattern is reinforced by the heatmap shown in Supplementary Fig. 1, which provides an integrated overview of direct and indirect functional effects across strains and highlights the high abundance of categories related to abiotic stress mitigation, motility, and chemotaxis. Consistently, genes involved in chemotaxis and motility (*tlpA*, *tlpC*, and *drcA*), stress response (*rhlE* and *nht*), and salinity and halotolerance (*yfbK*, *atpC*, and *atpD*) were detected in all genomes or in at least one copy per genome. The broadly conserved distribution of these genes, detailed in Supplementary Table S3, reinforces the genomic plasticity and ecological versatility of *Paenibacillus* species.

Mobile genetic elements (MGEs), including plasmids, ICEs, IMEs, and transposons, are key mediators of horizontal gene transfer (HGT) and can facilitate the dissemination of adaptive traits among bacterial populations (Dobrindt et al. 2004; Simpson et al. 2025). In this study, we observed that several genes associated with plant growth promotion and biocontrol were located on MGEs, particularly plasmids, which harbored a diverse set of genes involved in nodulation, IAA production, and other plant growth-promoting functions (Rodríguez-Beltrán et al. 2021; MacLean and San Millan 2019). The presence of these genes on MGEs highlights the potential of horizontal transfer to spread beneficial traits across *Paenibacillus* populations, reinforced by the detection of the same element in isolates from four different locations.

This genetic mobility may enhance the ecological versatility of strains, enabling adaptation to diverse environments and the establishment of important ecological interactions (Arnold et al. 2021). Furthermore, the use of strains containing MGEs as bioinoculants offers an additional advantage, as these bacteria could transfer beneficial traits to the resident soil microbiome without the need for reapplication, or even by utilizing only the MGEs from these strains (Brophy et al. 2018). Overall, our findings underscore the importance of considering MGE-associated genes when evaluating the ecological potential and biotechnological applicability of *Paenibacillus* strains.

The open configuration of the *Paenibacillus* pan-genome, supported by our findings, underscores the genus's genomic plasticity and its ongoing capacity to acquire novel genes from its environment. This genomic flexibility is vital for adaptation to the heterogeneous and competitive conditions of soil and rhizosphere ecosystems. A significant portion of both the core and accessory genomes is dedicated to functions associated with carbohydrate transport and metabolism (COG category G), reflecting an ecological strategy centered on nutrient acquisition and environmental sensing (Park and Kong 2018). Additionally, the remarkable biosynthetic potential for producing a wide array of bioactive secondary metabolites further enhances the genus's appeal for biotechnological innovation. These metabolites include antimicrobial compounds, siderophores, and phytohormones, which collectively support *Paenibacillus* in promoting plant growth, suppressing pathogens, and modulating plant immune responses.

## Conclusion

Our comprehensive comparative genomic study highlights *Paenibacillus* as a key microbial genus in plant–microbe interactions, with robust capabilities for promoting plant growth and suppressing phytopathogens through multifaceted mechanisms. The data presented herein emphasize the ecological relevance, genomic diversity, and biotechnological potential of this genus. These insights contribute to a deeper understanding of *Paenibacillus* biology and set the foundation for its application in sustainable agriculture. Future research should focus on isolating and characterizing novel *Paenibacillus* species, optimizing bioinoculant formulations, and evaluating their efficacy under field conditions. Furthermore, the impact of *Paenibacillus*-based products on native soil microbiota, ecosystem dynamics, and animal and human health warrants investigation. Altogether, this study supports the integration of *Paenibacillus* spp. into next-generation agricultural strategies aimed at reducing chemical inputs and enhancing crop productivity in a sustainable manner.

## Supplementary Information

Below is the link to the electronic supplementary material.

Supplementary file1 (PDF 33 KB)

Supplementary file2 (PDF 1798 KB)

Supplementary file3 (XLSX 1925 KB)

## Data Availability

The data are available in the manuscript.
