# Supplementary figures and images for: Comparative genomic analyses reveal key traits for biocontrol and the promotion of plant growth in Paenibacillus strains
Source: World J Microbiol Biotechnol. 2026 Feb 23;42(3):96. doi: 10.1007/s11274-026-04811-6 (PMC12926252; doi:10.1007/s11274-026-04811-6)

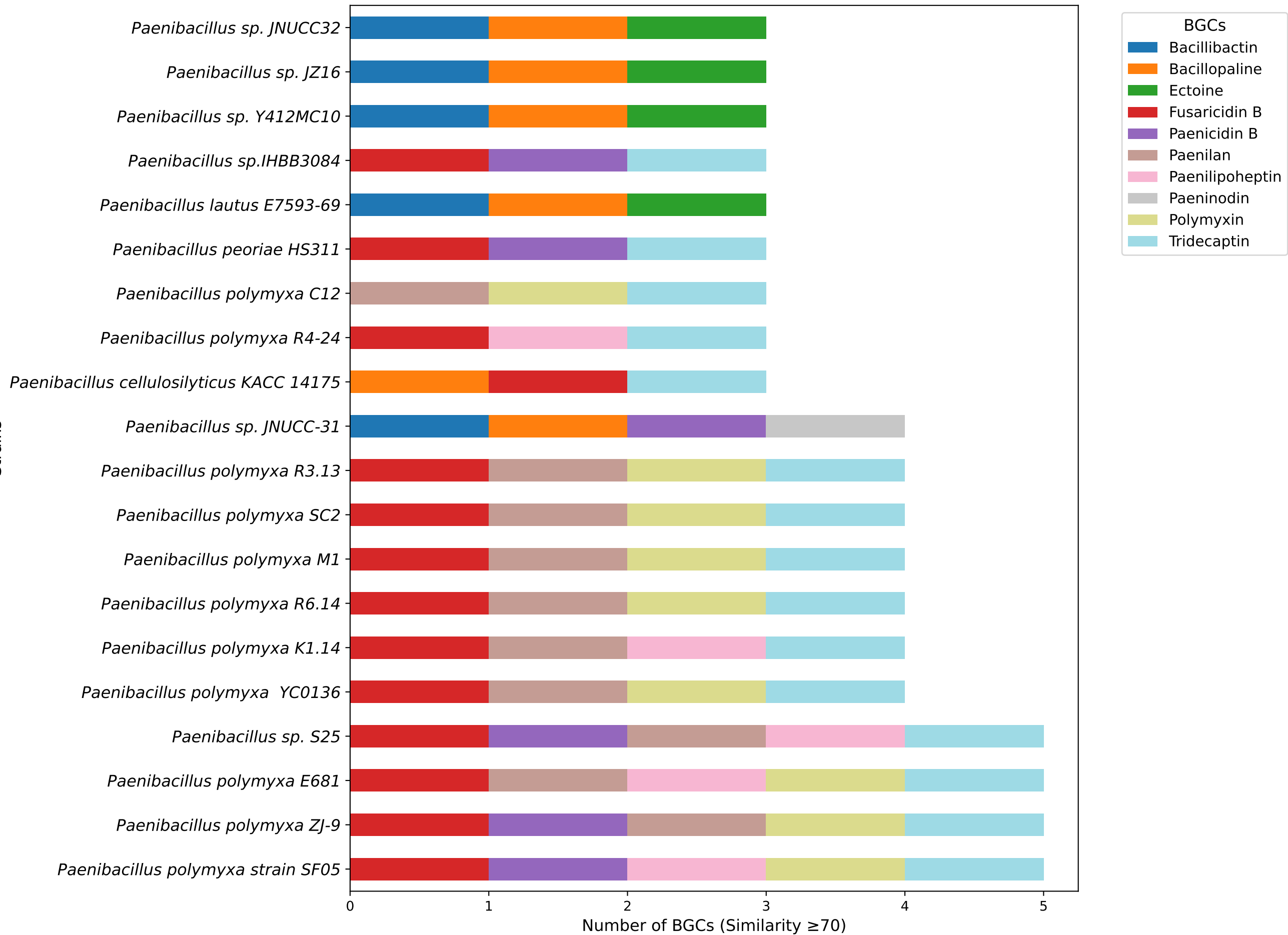

Supplement: Supplementary file 1 — Supplementary file1 (PDF 33 KB) [file 11274_2026_4811_MOESM1_ESM.pdf]
