# Supplementary material for: Comparative genomic analyses reveal key traits for biocontrol and the promotion of plant growth in Paenibacillus strains
Source: World J Microbiol Biotechnol. 2026 Feb 23;42(3):96. doi: 10.1007/s11274-026-04811-6 (PMC12926252; doi:10.1007/s11274-026-04811-6)
Supplement: Supplementary file 2 — Supplementary file2 (PDF 1798 KB) [file 11274_2026_4811_MOESM2_ESM.pdf]

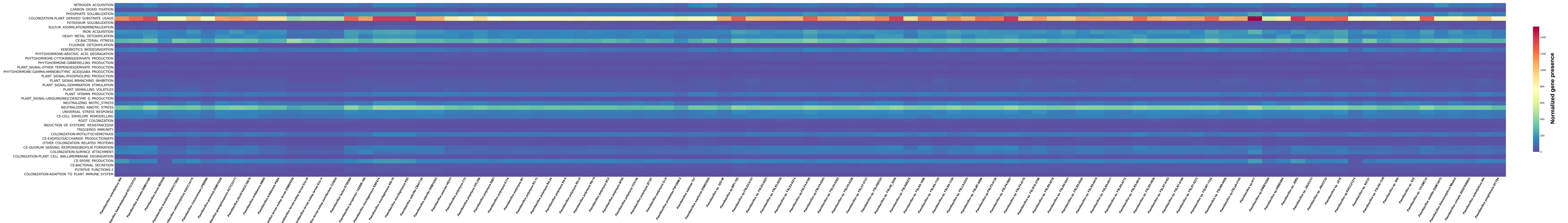
NITROGEN ACQUISITION
CARBON DIOXID FIXATION
PHOSPHATE SOLUBILIZATION
COLONIZATION-PLANT DERIVED SUBSTRATE USAGE
POTASSIUM SOLUBILIZATION
SULFUR ASSIMILATION|MINERALIZATION
IRON ACQUISITION
HEAVY METAL DETOXIFICATION
CE-BACTERIAL FITNESS
FLUORIDE DETOXIFICATION
XENOBIOTICS BIODEGRADATION
PHYTOHORMONE-ABSCISIC ACID DEGRADATION
PHYTOHORMONE-CYTOKININS|DERIVATE PRODUCTION
PHYTOHORMONE-GIBBERELLINS PRODUCTION
PLANT_SIGNAL-OTHER TERPENOID|DERIVATE PRODUCTION
PHYTOHORMONE-GAMMA-AMINOBUTYRIC ACID|GABA PRODUCTION
PLANT SIGNAL-PHOSPHOLIPID PRODUCTION
PLANT SIGNAL-BRANCHING INHIBITION
PLANT SIGNAL-GERMINATION STIMULATION
PLANT SIGNALLING VOLATILES
PLANT VITAMIN PRODUCTION
PLANT_SIGNAL-UBIQUINONE|COENZYME Q PRODUCTION
NEUTRALIZING BIOTIC_STRESS
NEUTRALIZING ABIOTIC STRESS
UNIVERSAL STRESS RESPONSE
CE-CELL ENVELOPE REMODELLING
ROOT COLONIZATION
INDUCTION OF SYSTEMIC RESISTANCE|ISR
TRIGGERED IMMUNITY
COLONIZATION-MOTILITY|CHEMOTAXIS
CE-EXOPOLYSACCHARIDE PRODUCTION|EPS
OTHER COLONIZATION RELATED PROTEINS
CE-QUORUM SENSING RESPONSE|BIOFILM FORMATION
COLONIZATION-SURFACE ATTACHMENT
COLONIZATION-PLANT CELL WALL|MEMBRANE DEGRADATION
CE-SPORE PRODUCTION
CE-BACTERIAL SECRETION
PUTATIVE FUNCTIONS-1
COLONIZATION-ADAPTION TO PLANT IMMUNE SYSTEM
Normalized gene presence
